# Lonidamine liposomes to enhance photodynamic and photothermal therapy of hepatocellular carcinoma by inhibiting glycolysis

**DOI:** 10.1186/s12951-023-02260-z

**Published:** 2023-12-15

**Authors:** Lei Lei, Wenbin Dai, Jiaping Man, Haitao Hu, Qiao Jin, Bo Zhang, Zhe Tang

**Affiliations:** 1 Department of Surgery, The Fourth Affiliated Hospital, International Institutes of Medicine, Zhejiang University School of Medicine, Yiwu, 322000 China; 2 MOE Key Laboratory of Macromolecule Synthesis and Functionalization of Ministry of Education, Department of Polymer Science and Engineering, Zhejiang University, Hangzhou, 310027 China; 3 Department of Surgery, The Second Affiliated Hospital, Zhejiang University School of Medicine, Hangzhou, 310058 China

**Keywords:** Lonidamine, Phototherapy, Glycolysis, Hypoxia, Heat shock proteins

## Abstract

Phototherapy, including photodynamic therapy (PDT) and photothermal therapy (PTT), has great promise in the treatment of cancer. However, there are many obstacles that can restrict the therapeutic efficacy of phototherapy. The hypoxic tumor microenvironment can restrict the production of reactive oxygen species (ROS) in PDT. As for PTT, the thermotolerance of cancer cells may lead to ineffective PTT. In this study, IR780 and glycolysis inhibitor lonidamine (LND)-encapsulated liposomes are prepared for photodynamic and photothermal therapy of hepatocellular carcinoma. IR780 can be used as a photosensitizer and photothermal agent for simultaneous PDT and PTT after being irradiated with 808 nm laser. LND can reduce the oxygen consumption of cancer cells by inhibiting glycolysis, which will relieve tumor hypoxia and produce more ROS for PDT. On the other hand, energy supply can be blocked by LND-induced glycolysis inhibition, which will inhibit the production of heat shock proteins (HSPs), reduce the thermotolerance of tumor cells, and finally enhance the therapeutic efficacy of PTT. The enhanced PTT is studied by measuring intracellular HSPs, ATP level, and mitochondrial membrane potential. The antitumor effect of IR780 and LND co-loaded liposomes is extensively investigated by in vitro and in vivo experiments. This research provides an innovative strategy to simultaneously enhance the therapeutic efficacy of PDT and PTT by inhibiting glycolysis, which is promising for future creative approaches to cancer phototherapy.

**Supplementary Information:**

The online version contains supplementary material available at 10.1186/s12951-023-02260-z.

## Introduction

Compared with traditional chemotherapy, phototherapy has many advantages, such as less invasiveness, high selectivity, and little cosmetic damage to the patients [1]. Because of these substantial advantages, phototherapy is considered as a viable strategy for cancer treatment. Now, phototherapy is being extensively researched, including photothermal therapy (PTT), photodynamic therapy (PDT), light-responsive molecule delivery, and light-controlled combination therapy [2, 3].

As a minimally invasive therapeutic strategy, PDT has gained widespread attention in the treatment of many superficial and confined tumors [4, 5]. PDT is an effective way to treat tumors, which can induce apoptosis in tumor cells by generating cytotoxic reactive oxygen species (ROS) [6–9]. Oxygen plays an important role in the efficacy of PDT. However, in the development of tumors, the oxygen supply from surrounding blood vessels cannot meet the huge demand for oxygen by tumor cells, leading to the hypoxic tumor microenvironment. Furthermore, with the consumption of oxygen in PDT, the oxygen available for the generation of cytotoxic ROS will be further reduced, which can strongly restrict the efficacy of PDT [10]. Many approaches were reported to overcome the limitations of PDT efficacy by enhancing oxygen supply [11]. For example, hemoglobin was used as the oxygen carrier to relieve the hypoxic microenvironment for enhanced photodynamic cancer therapy [12]. MnO_2_ is another important compound for the modulation of the tumor hypoxic microenvironment, which can induce the decomposition of H_2_O_2_ into oxygen [13, 14]. Reducing oxygen consumption by tumor cells might be another strategy to relieve the tumor hypoxic microenvironment [15, 16]. For example, cellular respiration can consume oxygen. Tumor hypoxia might be relieved by inhibiting cellular respiration, which might provide an important way to relieve tumor hypoxia. However, the relief of tumor hypoxia by reducing oxygen consumption is still rarely reported [17].

Photothermal therapy (PTT) is another widely adopted form of phototherapy, which can convert light energy into heat energy using photothermal agents after light irradiation. Localized hyperthermia can lead to the denaturation of cancer cell proteins, cell membrane damage, and other cytotoxic effects, which can lead to cell death [18–21]. However, in the practical application of PTT in treating tumors, relative high temperature (> 50 °C) at the tumor site is usually required, which will damage normal tissues near the tumor, causing a series of adverse effects. What’s more, the hyperthermia of tumor cells by PTT will stimulate their own repair mechanism and obtain tolerance to heat stress, which greatly reduces the efficacy of PTT. Therefore, there is an urgent need to reduce the thermal tolerance of tumor cells to achieve effective PTT. It has been found that the rapid production of heat shock proteins (HSPs) is widely observed in mammalian cells facing thermal stimuli [22]. HSPs are protective proteins that improve thermal tolerance and the self-stability of cells [23, 24]. The traditional method of inhibiting heat shock protein synthesis is mainly achieved by small molecular inhibitors (e.g., garcinic acid, 17-AAG, JG-98, etc.) or siRNA [25, 26]. However, most HSP inhibitors suffer from poor water solubility and high toxicity, while siRNAs are unstable and tend to have low delivery efficiency, limiting the application of these methods in the process of low-temperature photothermal therapy. Some methods were also reported to inhibit HSPs through gas therapy [27]. Since the production of HSPs is energy dependent, the inhibition of HSPs can also be achieved by reducing intracellular ATP production [28].

There are also many studies combining PDT and PTT for tumor treatment. The combination of PTT and PDT shows multiple advantages in improving the efficiency of tumor therapy [29, 30]. The combination of PDT and PTT inherits the advantages of low toxicity and side effects of phototherapy, and also enables the two treatment modalities to complement each other, which is an effective strategy to improve efficacy and reduce the side effects. In some studies, different photosensitizers and photothermal agents were integrated into one system. However, they are limited by the fact that they cannot be stimulated by the same light source to produce PDT and PTT effects [31]. The molecules that can be simultaneously used as photosensitizers and photothermal agents are more promising. For example, IR780 can absorb near-infrared (NIR) light, and subsequently produce both PDT and PTT effects under the stimulation of near-infrared light (808 nm) [32–34]. Compared with the commonly used near-infrared fluorescent dye, indocyanine green (ICG), IR780 shows better photostability and has a stronger fluorescence intensity, which deserves our attention [35, 36].

**Scheme 1:**
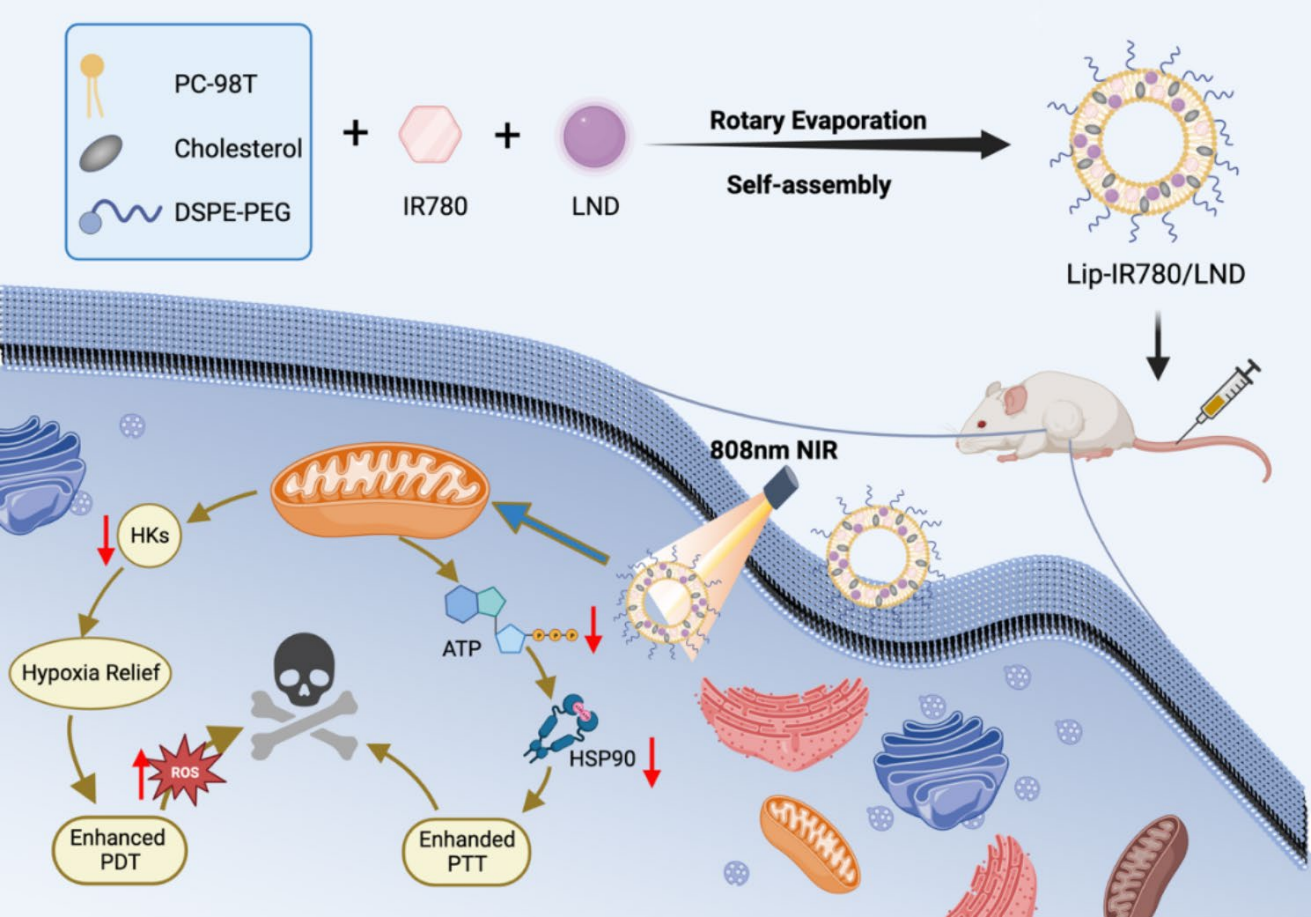
Schematic illustration of the preparation of Lip-IR780/LND and potential mechanism of LND enhanced PDT and PTT.

Herein, we put forward an innovative strategy to use the glycolysis inhibitor LND to enhance PDT and PTT of IR780 (Scheme 1). Due to the low toxicity and immunogenicity of liposomes, liposomes are used as drug delivery vehicles to encapsulate IR780 and LND. Lonidamine (LND) is an inhibitor of hexokinase and mitochondrial pyruvate carrier of glycolysis [37, 38]. LND might be used as a hypoxia ameliorator by reducing oxygen consumption, which can increase intracellular oxygen accumulation. Therefore, under the action of LND, more oxygen can be used as raw material to generate toxic ROS through PDT. As an important reducing agent in the body, glutathione (GSH) can consume ROS during PDT, which can restrict the therapeutic efficacy of PDT. Meanwhile, the decrease of GSH level by LND is also expected to improve PDT effect in cancer therapy. At the same time, LND is expected to inhibit cellular respiration to cut off energy production [39–41]. We intend to down-regulate the expression of HSPs in cancer cells by inhibiting the production of ATP, improving the therapeutic efficacy of PTT [42–44]. All these actions of LND may promote the production of ROS in PDT and simultaneously achieve reduced thermotolerance of tumor cells in PTT.

## Materials and methods

### Materials

Egg yolk lecithin (PC-98T) and cholesterol were obtained from AVT (Shanghai,China) Pharmaceutical Tech Co., Ltd. (Jiangsu, China). Methanol anhydrous, dichloromethane, and trichloromethane were obtained from Shanghai Sinopharm Chemical Reagent Co., Ltd. (Shanghai, China). Distearoyl phosphoethanolamine-PEG2000(DSPE-PEG_2000_) was purchased from Chongqing Yuying Pharmaceutical Technology Co., Ltd. (Chongqing, China). Fetal bovine serum (FBS) and Dulbecco’s Modified Eagle’s medium (DMEM) were bought from Gibco Life Technologies (USA). The high metastatic Human hepatocellular Carcinoma Cells (LM3) and Glutathione (GSH) test kit were obtained from Keygen Biotech Co., Ltd. (Jiangsu, China). Reactive oxygen species assay kit (DCFH-DA), ATP assay kit, and mitochondrial membrane potential assay kit (JC-10) were provided by Beyotime Biotechnology (Shanghai, China). Lonidamine, IR780, trypsin and CCK8 assay kit were provided by Meilun Biotechnology Co., Ltd (Jiangsu, China). Lactic acid detection kit was bought from Nanjing Jiancheng Bioengineering Institute (Nanjing, China). Oxygen Consumption Assay Kit was obtained from Shanghai Kanglang Biotechnology Co., Ltd. (Shanghai, China). Hexokinase activity assay kit was purchased from Abcam Plc. (USA). Hypoxia Inducible Factor 1 Alpha (HIF-1α) Kit and Hypoxyprobe-1 (HP-1) Kit were obtained from Hypoxyprobe (Burlington, USA). All reagents not mentioned were of analytical purity and used without further purification.

### Characterization

The hydrodynamic diameter and size distribution of the liposomes were characterized by dynamic light scattering (DLS) measurements (Zetasizer Nano-ZS, Malvern Instruments) equipped with a He-Ne laser (633 nm) at 25 ^◦^C. UV–vis spectra were measured by a UV-vis spectrometer (UV-2550, Shimadzu). The morphology of the nanoparticles was observed by cryo-transmission electron microscopy (cryo-TEM, Talos F200C, 200 kV). Fluorescence images were obtained via confocal laser scanning microscopy (CLSM) (Nikon, TS2-S-SM and Olympus, IX81).

### Synthesis of Lip-IR780/LND

Thin-film hydration method was used to prepare IR780-loaded liposomes (Lip-IR780), LND-loaded liposomes (Lip-IR780), and IR780 and LND co-loaded liposomes (Lip-IR780/LND). Lip-IR780 was first prepared. Egg yolk lecithin (90 mg), cholesterol (12 mg), DSPE-PEG_2000_ (6 mg), and IR780 (3 mg) were dissolved in 2 mL of chloroform. The above solution was rotationally evaporated at room temperature at 100 r/min under reduced pressure to allow sufficient evaporation of the organic solvent. A thin layer of IR780-loaded liposome film was then obtained. Subsequently, 5 mL of ultrapure water was added to the evaporated-off conical flask and then sonicated. The solution was then filtrated to obtain Lip-IR780. In order to prepare Lip-LND, egg yolk lecithin (90 mg), cholesterol (9 mg), DSPE-PEG_2000_(6 mg), and LND (3 mg) were dissolved in 1 mL chloroform and 1 mL methanol. The same method as above was used to obtain Lip-LND. Finally, in order to prepare Lip-IR780/LND, egg yolk lecithin (90 mg), cholesterol (12 mg), DSPE-PEG_2000_ (6 mg), LND (3 mg), and IR780 (1.5 mg) were dissolved in 1 mL methanol and 1 mL chloroform. The same method as above was used to obtain Lip-IR780/LND.

### Morphology of liposomes

The solutions of the samples were spotted onto copper grids to form a very thin liquid layer. Then the layer was rapidly frozen in liquid ethane. The morphology of the drug-loaded liposomes was observed by cryo-TEM. Lip-IR780, Lip-LND and Lip-IR780/LND at a concentration of 15 µg mL^− 1^ were used to display the morphologies under cryo-TEM.

### Size stability and zeta-potential measurement

Lip-IR780, Lip-LND, and Lip-IR780/LND were incubated in PBS for 7 days. On days 1, 3, 5, and 7, the liposomal solution was used to measure the hydrodynamic diameter by Zetasizer Nano-ZS at 25 ℃. In order to measure zeta-potential, the liposomes were incubated in PBS and the zeta-potential was also measured with Zetasizer Nano-ZS.

### Cell culture

LM3 cells were cultured in Dulbecco’s modified Eagle medium (DMEM, Wisent, Nanjing, China) containing 10% FBS, 100 UmL^− 1^ penicillin, and 100 mg mL^− 1^ streptomycin in an incubator (Thermo Fisher, Waltham, USA) at 37 °C with 5% CO2.

### Cellular uptake studies

To observe the cellular uptake behaviors of Lip-IR780, Lip-LND, and Lip-IR780/LND, confocal laser scanning microscopy (CLSM) was applied. LM3 cells were seeded into 24-well plates in DMEM at 4 × 10^4^ cells per well. After cells were incubated for 24 h, the culture medium was replaced by fresh medium containing Lip-IR780, Lip-LND, and Lip-IR780/LND (equal concentration IR780 7.5 µg mL^− 1^ and LND 15 µg mL^− 1^). LM3 cells were further cultured for another 4 h. After washing the LM3 cells with PBS three times, the fluorescent images were captured by fluorescent microscope.

### Cytotoxicity assay in vitro

The cytotoxicity of three liposomes were evaluated by cell counting kit-8 (CCK-8) assay using LM3 cells in culture media. LM3 cells were seeded into 96-well plates at 8 × 10^3^ cells per well in DMEM (200 µL). After cells were incubated for 24 h, Lip-IR780, Lip-LND, and different concentrations of Lip-IR780/LND were added and incubated for 5 h. The medium was then replaced with fresh medium, and the cells were irradiated with a NIR laser (4 w, 5 min). Then, cells were incubated for another 24 h. The culture medium was replaced with DMEM medium containing 10% CCK-8 and further cultured at 37 ◦C for another 4 h. The absorbance of formazan was determined by a microplate reader (BioTek, Synergy H1) at 450 nm. Quantitative data was rendered as average ± SD (n = 6).

### GSH depletion in vitro

The GSH test kit (Keygen KGT006) was used to measure the concentration of GSH in vitro. LM3 cells were seeded into 6-well plates at 1 × 10^6^ cells per well in DMEM medium and cultured for 24 h. The culture medium was substituted with fresh medium containing Lip-IR780, Lip-LND, and Lip-IR780/LND (equal concentration IR780 7.5 µg mL^− 1^ and LND 15 µg mL^− 1^). Then trypsinization was used to harvest cells after 4 h incubation. According to the manufacturer’s directions, the concentration of GSH was measured by the UV-vis spectrum at 410 nm, and GSH concentration was calculated by the following Eq. (1), where A is the absorbance and C is the concentration.

1 $${\text{C}}_{\text{G}\text{S}\text{H}} =({\text{A}}_{\text{m}\text{e}\text{a}\text{s}\text{u}\text{r}\text{e}\text{d}}-{\text{A}}_{\text{b}\text{l}\text{a}\text{n}\text{k}}) / ({\text{A}}_{\text{s}\text{t}\text{a}\text{n}\text{d}\text{a}\text{r}\text{d}}-{\text{A}}_{\text{b}\text{l}\text{a}\text{n}\text{k}})\times {\text{C}}_{\text{G}\text{S}\text{H}\text{s}\text{t}\text{a}\text{n}\text{d}\text{a}\text{r}\text{d}}$$

### Intracellular ROS quantification

LM3 cells (8 × 10^4^ cells) were seeded into 24-well plates in DMEM (500 µL) with 10% FBS. After 24 h incubation, the culture medium was substituted with fresh medium containing Lip-IR780, Lip-LND, and Lip-IR780/LND (equal concentration IR780 7.5 µg mL^− 1^ and LND 15 µg mL^− 1^). After 24 h incubation, the medium was replaced with fresh medium containing ROS probe (2’,7’-dichlorofluorescin diacetate, DCFH-DA, 10 µM), and the cells were irradiated with a NIR laser (0.4 W cm^− 2^, 5 min). After 4 h, the cells were washed three times with PBS and imaged with confocal laser scanning microscopy.

### Mitochondrial membrane potential assay in vitro

Mitochondrial membrane potential kit JC-10 (Beyotime, China) was used to evaluate the membrane potential of mitochondria. 4 × 10^4^ LM3 cells were seeded into 24-well plates in DMEM for 24 h. The medium was replaced with fresh medium containing Lip-IR780, Lip-LND, and Lip-IR780/LND (equal concentration IR780 7.5 µg mL^− 1^ and LND 15 µg mL^− 1^), respectively, for 4 h. Then the cells were further cultured with JC-10 solution for another 20 min at 37℃. Subsequently, the cells were washed with PBS three times and observed by confocal laser scanning microscopy.

### Measurement of ATP level in vitro

The ATP assay kit (Beyotime, China) was used to evaluate the ability of LND to inhibit the synthesis of ATP. LM3 cells were seeded into 24-well plates at 4 × 10^4^ per well and incubated for 24 h. The cells were then treated with different concentrations of Lip-IR780 and Lip-LND for 12 h. The intracellular ATP level was evaluated via the ATP assay kit. What’s more, the fluorescence signals were determined by chemiluminescence, and the relative intracellular ATP level was calculated using the following Eq. (2):

2 $$\text{R}\text{e}\text{l}\text{a}\text{t}\text{i}\text{v}\text{e}\,\text{A}\text{T}\text{P}\,\text{l}\text{e}\text{v}\text{e}\text{l} \left(\text{\%}\right) = ({\text{F}}_{\text{s}\text{a}\text{m}\text{p}\text{l}\text{e}}-{\text{F}}_{\text{b}\text{l}\text{a}\text{n}\text{k}}) / ({\text{F}}_{\text{c}\text{o}\text{n}\text{t}\text{r}\text{o}\text{l}}-{\text{F}}_{\text{b}\text{l}\text{a}\text{n}\text{k}}) \times 100$$

where F_sample_ is the fluorescence signal of the group of samples, F_blank_ is the fluorescence signal of the blank group, and F_control_ is the negative control group.

### Measurement of extracellular lactate in vitro

Lactic acid detection kit (JianCheng Bioengineering Institute, Nanjing) was used to measure extracellular lactate concentration. LM3 cells were seeded into 24-well plates at 4 × 10^4^ per well and incubated for 24 h. LM3 cells were treated with different concentrations of Lip-IR780 and Lip-LND for 12 h. After that, the culture medium was collected. According to the manufacturer’s instructions, lactate concentration was detected using a microplate Bio-Rad reader at 450 nm.

### Measurement of oxygen consumption in vitro

In order to assess the oxygen consumption rate (OCR), Oxygen Consumption Assay Kit (Shanghai Kanglang Biotechnology Co., Ltd.) was used. LM3 cells were seeded into 96-well plates at 1 × 10^4^ per well and incubated for 24 h. Different concentrations of Lip-IR780 and Lip-LND were then treated for 12 h. The oxygen consumption rate was detected according to the manufacturer’s instructions.

### Measurement of hexokinase in vitro

For the detection of hexokinase, LM3 cells were seeded into 24-well plates at 1 × 10^4^ per well for 24 h. LM3 cells were treated with different concentrations of Lip-IR780 and Lip-LND for 12 h later. Then cells were harvested and washed three times with PBS. According to the manufacturer’s instructions, Hexokinase activity assay kit (ab211103, Abcam) was used to measure the activity of hexokinase.

### Live/Dead staining in vitro

Firstly, LM3 cells were seeded into 24-well plates at 100 × 10^3^cells per well in DMEM (1 mL). Then, LM3 cells were incubated with Lip-IR780 (7.5 µg mL^− 1^), Lip-LND (15 µg mL^− 1^), and Lip-IR780/LND (LND, 15 µg mL^− 1^, IR780, 7.5 µg mL^− 1^). The groups with light were irradiated (0.4 W cm^− 2^, 5 min) 5 h later. After 24 h, the aspirated supernatant from the cell wells was placed into a 15 mL centrifuge tube to be used as a reserve and to mark each group. Then, trypsin (Meilun, Dalian, China) was used to digest the adherent cells in the well plates for 2 min. After that, cells were aspirated and mixed in the corresponding centrifuge tubes. The supernatant was discarded, and the cell precipitate was washed three times with PBS after centrifugation (1000r/5min). Then cells were transferred to 48-well plates. Stained with calcein O,O′-diacetate tetrakis (acetoxymethyl) ester (Calcein-AM) and propidium iodide (PI) according to the manufacturer’s instructions and incubated for 30 min in the dark. Finally, the prepared cells were photographed by an inverted fluorescence microscope (Nikon Ti2, Japan).

### Western blot analysis

The intracellular HSP90 level after different treatments was detected by Western blot assay. LM3 cells were seeded into 6-well plates at 1 × 10^5^ cells per well in DMEM. After 24 h incubation at 37 °C under 5% CO_2_, the cells were treated with PBS, Lip-IR780, Lip-LND, and Lip-IR780/LND (LND, 15 µg mL^− 1^, IR780, 7.5 µg mL^− 1^) for another 4 h, respectively. After washing the cells with PBS for three times, the cells treated with Lip-IR780 and Lip-IR780/LND were exposed with 808 nm laser (0.4 W cm^− 2^, 5 min). After washing the cells with PBS for three times, the cells were harvested by trypsinization. The total protein was quantified using BCA Protein Quantification Kit. After transferring onto the polyvinylidene fluoride membrane, 10% sodium dodecyl sulfate polyacrylamide gel electrophoresis (SDS-PAGE) was used to separate the proteins of each sample. The membrane was incubated with T-TBS containing 5% bovine albumin (BSA) for 1 h and then incubated with relevant primary antibody Anti-HSP90 at 4 °C overnight. Then, the membranes were washed with T-TBS for three times and 12 hybridized with Goat anti-Rabbit IgG (H + L) as secondary antibody against HSP90 primary antibody at 25℃ for 1 h. The membranes were visualized on X-ray films and detected by chemiluminescence using SuperSignal® West Dura Extended Duration Substrate.

### Antitumor effect and biosafety of drug-loaded liposomes in vivo

All animal experiments were conducted following the guidelines for Institutional Animal Care and Use Committee, Zhejiang Center of Laboratory Animals (ZJCLA) and the “Principles of Laboratory Animal Care” (NIH publication no.86 − 23, revised 1985). The assigned approval number was ZJCLA-IACUC-20,010,251. The authors state that the animal experiments conformed with the Helsinki Declaration of 1975, as revised in 2008 (5) concerning Human and Animal Rights. The antitumor efficacy of the liposomal nanodrugs was evaluated on subcutaneous LM3 xenograft tumor models. Healthy male BALB/c nude mice (20 ± 2 g, 4 ~ 5 weeks old) were purchased from Zhejiang Academy of Medical Sciences. LM3 cells (1 × 10^6^) were injected subcutaneously into the right flank of mice. When the tumors reached about 100 mm^3^, the mice were randomly divided into five groups (Saline, Lip-IR780, Lip-LND, Lip-IR780 + L, and Lip-IR780/LND). Lip-IR780 (7.5 µg mL^− 1^), Lip-LND (15 µg mL^− 1^), and Lip-IR780/LND (LND, 15 µg mL^− 1^, IR780, 7.5 µg mL^− 1^) were injected into the tail vein. Five h later, the groups with light were locally irradiated by 808 nm laser (0.4 W cm^− 2^) for 5 min. The tumor volume and body weight of mice were calculated and recorded regularly to assess the anti-tumor efficacy of drugs. After 18 days, the mice were euthanized, and the tumors of each group were weighted and recorded by a digital camera. Blood tests and H&E were used to verify the biosafety of nanodrugs.

The tumor volume was calculated according to the following Eq. (3):

3 $$\text{V}\text{o}\text{l}\text{u}\text{m}\text{e} = \left(\text{T}\text{u}\text{m}\text{o}\text{r}\,\text{L}\text{e}\text{n}\text{g}\text{t}\text{h}\right) \times {\left(\text{T}\text{u}\text{m}\text{o}\text{r} \text{W}\text{i}\text{d}\text{t}\text{h}\right)}^{2}/2$$

### Body imaging and biodistribution in vivo

LM3 subcutaneous tumor-bearing nude mice were divided into two groups randomly and injected with free-IR780 and Lip-IR780, respectively. At the predetermined time, in vivo body fluorescent images were taken by an in vivo imaging system by collecting the IR780 signals.

### Pharmacokinetic studies in vivo

To obtain the pharmacokinetics profiles, ten tumor-free ICR mice were divided into two groups and intravenously injected with free-IR780 and Lip-IR780, respectively. At different time points (0.033, 0.5, 1, 3, 6, 12, and 24 h), 100 µL of blood was collected. The plasma was obtained after 2000 rpm for 15 min and diluted with 1 × PBS (100 µL). Then, the fluorescence intensity of IR780 was measured.

### Thermal imaging in vivo

To investigate the photothermal properties of liposomal drugs, LM3 subcutaneous tumor-bearing nude mice were used. Lip-IR780/LND was injected *via* tail vein. The changes of temperature at the tumour mass were observed for different time points after irradiated by 808 nm laser at 0.4 W cm^− 2^ using the infrared thermal imager(FLIR E4).

### Statistics analysis

The data were presented as mean ± standard deviation (SD). Making use of GraphPad Prim 9.5.0 for statistical analysis. Student’s t test was used to determine significant differences between groups. *p < 0.05 was considered a significant difference and ***p < 0.001 was considered a highly significant difference.

## Result and discussion

### Preparation and characterization of drug-loaded liposomes

**Fig. 1:**
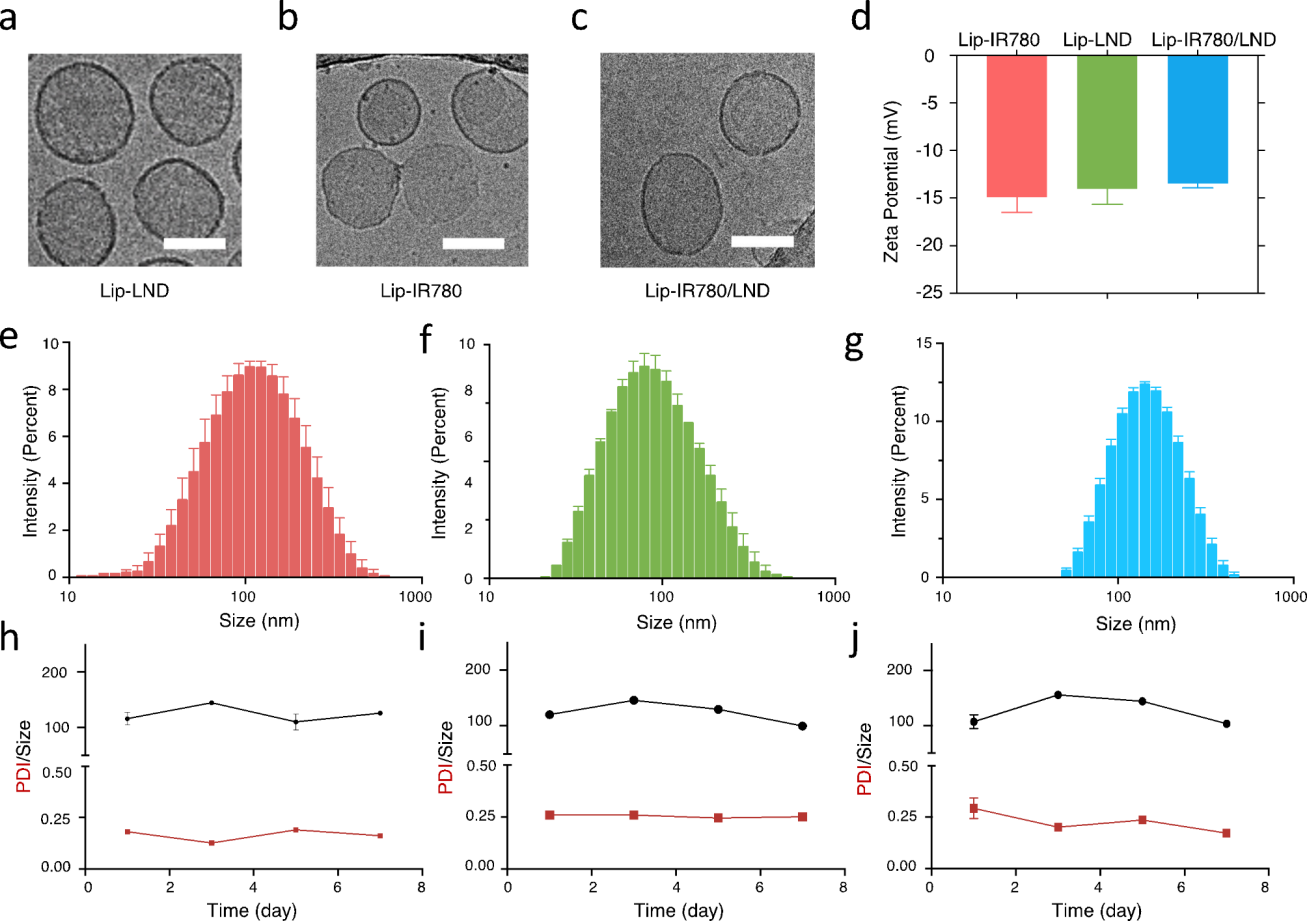
Characterization of drug-loaded liposomes. (**a-c**) The Cryo-TEM images of Lip-IR780, Lip-LND, and Lip-IR780/LND. Scale bar: 100 nm. (**d**) Zeta potential of Lip-IR780, Lip-LND, and Lip-IR780/LND. (**e-g**) Hydrodynamic diameters of Lip-IR780, Lip-LND, and Lip-IR780/LND. (h-i) Hydrodynamic diameters (upper) and PDI changes (lower) of Lip-IR780, Lip-LND, and Lip-IR780/LND upon incubating in PBS for 7 days

Since liposome is the most widely accepted nanodrug in market, IR780 and LND were encapsulated into liposomes in this research. The drug encapsulated liposomes, Lip-IR780, Lip-LND, and Lip-IR780/LND, were prepared by the classical lipid film hydration method. The drug loading content (DLC) and entrapment efficiency (EE) of Lip-IR780, Lip-LND, and Lip-IR780/LND were calculated and the results were shown in Table S1. To confirm the formation of liposomal nanodrugs, the morphologies were revealed by cryo-transmission electron microscopy (cryo-TEM). All of the liposomes exhibited monolayered liposomal structures with a diameter of approximately 100 nm (Fig. 1a-c). The surface charge of the liposomes was then studied by measuring zeta potential. As shown in Fig. 1d, the zeta potential of Lip-IR780, Lip-LND, and Lip-IR780/LND were − 14.9 mV, -14.0 mV, and − 13.2 mV, respectively. Furthermore, the intensity average hydrodynamic diameters of Lip-IR780, Lip-LND, and Lip-IR780/LND were 123.9 nm, 103.0 nm, and 134.8 nm, respectively, measured by dynamic light scattering (DLS), as shown in Fig. 1e-g. Thus, we demonstrated that drug-loaded liposomes with the size of about 100 nm were successfully prepared, which can be used for systemic drug delivery via blood transport and also for passive targeting of tumors via the enhanced permeability and retention effect (EPR) effect.

The excellent stability of nanoparticles is a key requirement for their systemic intravenous administration. We then determined the stability of liposomal nanodrugs by measuring the size of the three nanodrugs in PBS using DLS consecutively. The results of DLS showed that the liposomal nanodrugs did not undergo significant particle size changes, and the polydispersity index (PDI) was almost unchanged after 7 days’ incubation (Fig. 1h-j). These results demonstrated that the dug-loaded liposomes prepared by this method exhibited good stability.

**Fig. 2:**
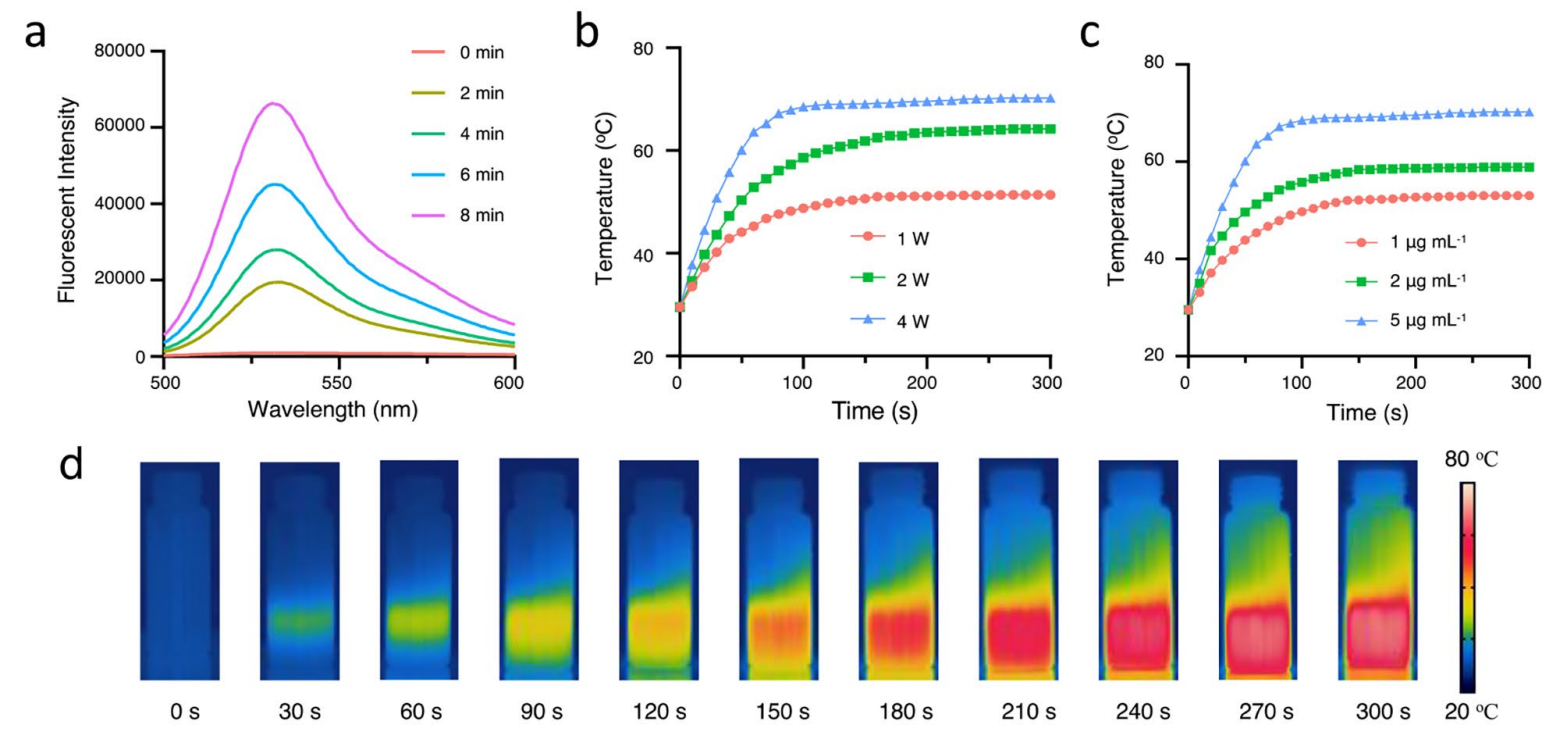
Photodynamic and photothermal effect of drug loaded liposomes in vitro. (**a**) The generation of ROS was detected by fluorescent spectrum after Lip-IR780 was irradiated by 808 nm laser for different illumination time (0, 2, 4, 6, 8 min). DCFH-DA was used as an ROS fluorescent probe. (**b**) Temperature of Lip-IR780 solution under 808 nm laser irradiation with different exposure intensities (0.1, 0.2, 0.4 W cm − 2). (**c**) Temperature elevation of Lip-IR780 at different concentrations (1, 2, 5 µg mL^− 1^) under 808 nm laser irradiation. (**d**) Temperature changes of Lip-IR780/LND after laser irradiation were recorded by infrared thermography

IR780 was used as a photosensitizer and photothermal agent simultaneously in this research. The photodynamic effect of Lip-IR780 was investigated at first. 2′,7′-dichlorodihydrofluorescein diacetate (DCFH-DA) was used as an ROS fluorescent probe to evaluate the ability of Lip-IR780 to generate ROS after laser irradiation since DCFH-DA can be oxidized to 2′,7′-dichlorodihydrofluorescein (DCFH) by ROS. As shown in Fig. 2a, the fluorescence intensity increased significantly with the increase of laser irradiation time, which indicated that Lip-IR780 could effectively generate ROS after 808 nm laser irradiation. The photothermal effect of Lip-IR780 was then studied. A significant increase of solution temperature was observed after Lip-IR780 solution was exposed to 808 nm laser irradiation. As the light intensity increased, the final temperature and heating rate increased accordingly, indicating the generation of heat can be well controlled by the light intensity (Fig. 2b). Meanwhile, by comparing the photothermal effect of Lip-IR780 with different concentrations under the same light intensity, a higher concentration of IR780 induced a higher final temperature and a faster heating rate (Fig. 2c). In addition, the increase of solution temperature after laser irradiation was also visually observed in infrared thermograms (Fig. 2d). All these results indicated that IR780 could be used as an excellent photosensitizer and photothermal agent.

### LND-induced enhancement of PDT in vitro

Liposomes should be uptaken by cells to play the role. The internalization of drug-loaded liposomes by LM3 cells was first investigated by fluorescent microscopy. Although LND is non-fluorescent, IR-780 can be used as a fluorescent probe to study the internalization of liposomes. As shown in Fig. S1, strong red fluorescence of IR780 could be observed after LM3 cells were incubated with Lip-IR780 and Lip-IR780/LND, indicating efficient internalization of liposomes by LM3 cells. Meanwhile, a time-dependent increase in intracellular red fluorescence was observed (Fig. S1).

**Fig. 3:**
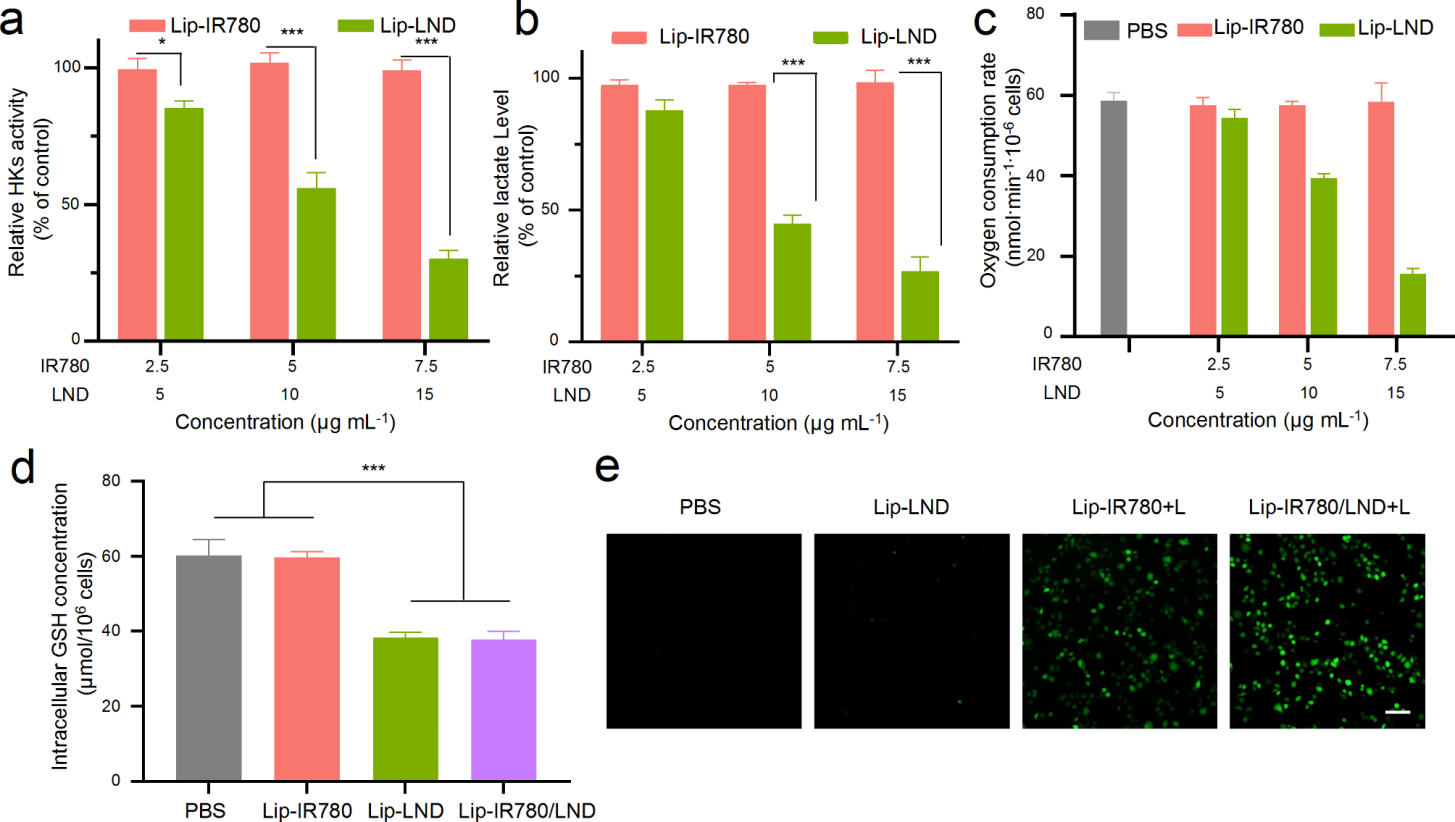
The enhanced PDT of Lip-IR780/LND in vitro. (**a**) The intracellular HKs level after LM3 cells were treated with different concentrations of Lip-IR780 or Lip-LND. (**b**) The level of extracellular lactate content after LM3 cells were treated with different concentrations of Lip-IR780 or Lip-LND. (**c**) The inhibition of intracellular oxygen consumption after LM3 cells were treated with different concentrations of Lip-IR780 or Lip-LND. (**d**) The intracellular GSH concentration after LM3 cells were treated with PBS, Lip-IR780, Lip-LND, and Lip-IR780/LND. (**e**) The production of ROS detected by DCFH-DA in LM3 cells. The cells were treated with PBS, Lip-IR780, Lip-LND + L, and Lip-IR780/LND + L. Scale bar: 100 μm. (n = 5). Data were expressed as means ± SD. ^*^p < 0.05, ^**^p < 0.01, ^***^p < 0.001

LND is an anticancer drug that interferes with the energy metabolism of cancer cells, principally inhibiting glycolysis, by its inhibition effect of mitochondrially-bound hexokinase (HK). As HK-II is the first key enzyme involved in glycolysis, it can be used as an indicator to evaluate the rate of glycolysis. As an HK inhibitor, LND can block energy supply by inhibiting glycolysis, so Lip-LND might ultimately affect intracellular oxygen consumption. After incubation of Lip-IR780 and Lip-LND with LM3 cells for 24 h, the relative intracellular HK level was evaluated by hexokinase activity assay kit. As shown in Fig. 3a, the intracellular HK level almost did not change after incubating with different concentrations of IR780. However, if LM3 cells were incubated with Lip-LND, the intracellular HK level could be significantly reduced, suggesting the excellent inhibitory effect of LND on HK activity. During glycolysis, glucose undergoes a series of oxidative phosphorylation processes to produce lactose and pyruvate, which can then enter the mitochondria and participate in oxygen production via the tricarboxylic acid cycle. Therefore, the inhibition of glycolysis can be assessed by measuring lactate level. Lactate level was measured using a lactate assay kit to determine the extracellular lactate level after different treatments. As shown in Fig. 3b, the extracellular lactate content was not affected after incubation with Lip-IR780. When LM3 cells were treated with Lip-LND, the extracellular lactate content decreased with increasing LND concentration, indicating that LND could effectively inhibit glycolysis. The reduction of lactate can further inhibit energy production in the mitochondrial respiratory chain. The oxygen consumption rate (OCR) of LM3 cells after different treatments was also measured using the OCR assay kit (Fig. 3c). As consistent with the above results, the OCR of LM3 cells could be effectively reduced after incubation with Lip-LND. To sum up, Lip-LND could inhibit glycolysis to reduce oxygen consumption by inhibiting HK activity, which might increase intracellular oxygen accumulation. Since oxygen is the raw material in PDT, the integration of LND in PDT might relieve tumor hypoxia to enhance the therapeutic efficacy of PDT.

GSH is an important intracellular antioxidant that can consume ROS to restrict the therapeutic efficacy of PDT. It is an effective way to improve the therapeutic efficacy of PDT by reducing intracellular GSH level. The influence of Lip-LND on intracellular GSH level was then evaluated. As shown in Fig. 3d, intracellular GSH level was unaffected by Lip-IR780. In contrast, the intracellular GSH level was significantly reduced after incubating LM3 cells with Lip-LND or Lip-IR780/LND, which implied that LND could reduce the intracellular GSH level. The decrease of intracellular GSH level by LND was advantageous for photodynamic cancer therapy.

The intracellular photodynamic effect of IR780 with or without LND was then investigated after 808 nm laser irradiation. Intracellular ROS production was detected after incubating LM3 cells with Lip-LND, Lip-IR780 with laser irradiation (Lip-IR780 + L), and Lip-IR780/LND with laser irradiation (Lip-IR780/LND + L) using DCFH-DA as the ROS probe (Fig. 3e). The intracellular green fluorescence of the ROS probe was barely observed after Lip-LND treatment as observed microscopically. This is because even though LND could lead to a decrease of intracellular oxygen consumption and an increase in oxygen accumulation, ROS cannot be produced in the absence of photosensitizers. However, strong green fluorescence was observed after LM3 cells were treated with Lip-IR780 + L or Lip-IR780/LND + L, which indicated the production of ROS due to the photodynamic effect of IR780. Interestingly, Lip-IR780/LND-treated cells exhibited stronger green fluorescence than Lip-IR780-treated cells, suggesting that LND could increase the production of ROS intracellularly, which implied that LND could enhance the photodynamic effect of IR780.

### Improving the efficiency of PTT by LND in vitro

**Fig. 4:**
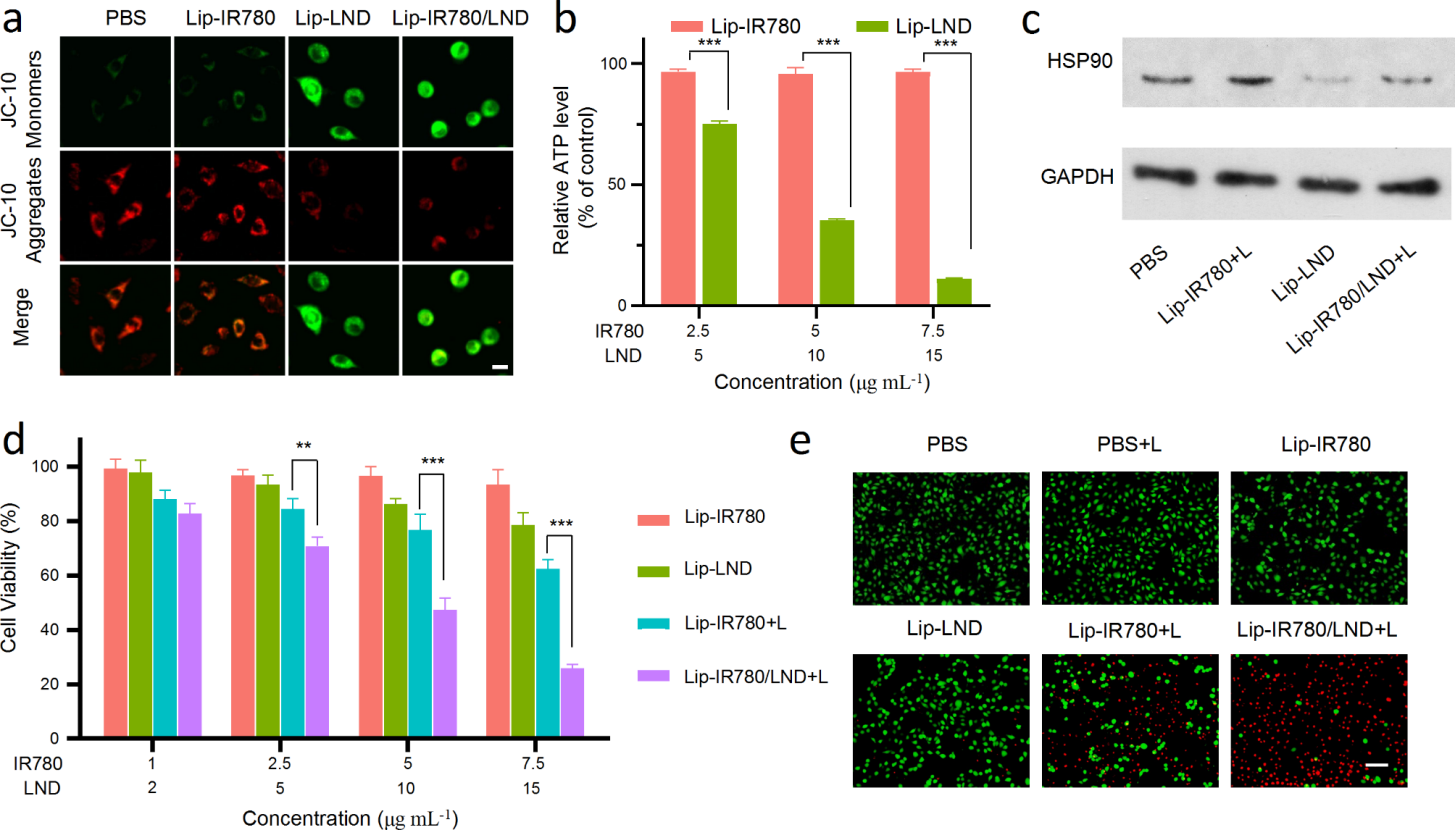
The enhanced PTT and cytotoxicity of Lip-IR780/LND in vitro. (**a**) CLSM images of LM3 cells under JC-10 staining after incubation with PBS, Lip-IR780 + L, Lip-LND, and Lip-IR780/LND + L. Scale bar: 5 μm (**b**) Relative ATP level in LM3 cells after treatment with Lip-IR780 and Lip-LND for 12 h. (**c**) The detection of intracellular HSP90 level by Western blot assay after LM3 cells were received with PBS, Lip-IR780 with laser irradiation (Lip-IR780 + L), Lip-LND, and Lip-IR780/LND with laser irradiation (Lip-IR780/LND + L). (**d**) Relative viability of LM3 cells after treatment with Lip-IR780, Lip-LND, Lip-IR780 + L, and Lip-IR780/LND + L. (**e**) In vitro cytotoxicity of different treatments (PBS, PBS + L, Lip-IR780, Lip-LND, Lip-IR780 + L, and Lip-IR780/LND + L) against LM3 cells was measured by Live/Dead double staining. The concentrations of Lip-IR780 and Lip-LND were 7.5 µg mL^− 1^ and 15 µg mL^− 1^, respectively. Scale bar: 100 μm. (n = 5). Data were expressed as means ± SD. ^*^p < 0.05, ^**^p < 0.01, ^***^p < 0.001

As discussed above, LND could promote photodynamic effect by inhibiting cellular respiration. Cellular respiration is coupled to energy production in mitochondria. We elaborated on the effect of LND on mitochondrial energy production. Firstly, mitochondrial integrity was evaluated by observing the mitochondrial membrane potential (MMP) using the JC-10 mitochondrial membrane potential assay kit (Fig. 4a). Under normal conditions, JC-10 assumes an aggregated form (JC-aggr) and accumulates in the mitochondrial matrix with red fluorescence. However, if the mitochondria are damaged and the membrane potential is lowered, JC-10 assumes a monomeric form (JC-mono) and appears to green fluorescence. After 24 h incubation of LM3 cells with Lip-IR780, JC-10 aggregated and showed red fluorescence under confocal fluorescence microscopy, suggesting intact mitochondrial membranes and healthy mitochondria. In contrast, LM3 cells incubated with Lip-LND and Lip-IR780/LND for 24 h showed more intense green fluorescence of JC-10 monomers. The results suggested that LND-loaded liposomes can disrupt mitochondrial integrity.

HSPs are key initiating proteins in the heat-resistant defense mechanisms triggered by tumors; thus, the overexpression of HSPs severely limits the anti-tumor efficacy of PTT. The production of HSPs is dependent on ATP provided by mitochondria. Intracellular ATP level after different treatments were then measured using the ATP kit. As shown in Fig. 4b, Lip-IR780 had no effect on intracellular ATP production. However, the intracellular ATP level was significantly reduced after LM3 cells were incubated with Lip-LND, indicating that Lip-LND could effectively block energy supply. HSP90 is one of the most widely studied heat shock proteins. The expression of HSP90 after incubation of LM3 cells with Lip-IR780, Lip-LND, and Lip-IR780/LND was subsequently investigated by Western blot. As shown in Fig. 4c and S3, the expression of HSP90 was unaffected after incubation of LM3 cells with Lip-IR780. In contrast, the expression of HSP90 was significantly reduced after incubation with Lip-LND and Lip-IR780/LND, indicating that LND could effectively inhibit the expression of HSP90 and increase the heat sensitivity of tumor cells.

After confirming the multiple roles of LND in reducing oxygen consumption, down-regulating intracellular GSH level, and inhibiting HSPs expression, we further investigated whether LND could improve the therapeutic effect of IR780-triggered phototherapy. To demonstrate the therapeutic potential of LND in phototherapy, cell viability after different treatments was examined using the CCK-8 assay. As shown in Fig. 4d, LM3 cells incubated with Lip-LND exhibited weak proliferation inhibition, indicating that inhibition of glycolysis by LND alone is not sufficient to inhibit the proliferation of cancer cells. The viability of LM3 cells was as high as 80% after incubating with 15 µg mL^− 1^ Lip-LND (LND equivalent). Meanwhile, Lip-IR780 didn’t show an obvious inhibitory effect on the proliferation of LM3 cells in the absence of light irradiation. In contrast, Lip-IR780 could inhibit the proliferation of LM3 cells under 808 nm laser irradiation. The viability of LM3 cells was about 60% after incubating with 7.5 µg mL^− 1^ Lip-IR780 (IR780 equivalent) under laser irradiation. When LM3 cells were treated with Lip-IR780/LND under 808 nm laser irradiation, the combined therapy group was found to reduce the survival rate of cancer cells to about 20%, achieving a powerful anti-tumor therapy. The remarkable proliferation inhibition ability of Lip-IR780/LND under laser irradiation might be attributed to the excellent glycolysis inhibitory effect of LND. LND could improve the therapeutic efficacy of photodynamic and photothermal therapy by reducing oxygen consumption, down-regulating intracellular GSH level, and inhibiting HSPs expression. Furthermore, the excellent phototherapy efficacy of Lip-IR780/LND under laser irradiation was reconfirmed by live/dead staining (Fig. 4e). Firstly, the untreated group was unable to induce cell death with or without laser irradiation, demonstrating that light alone didn’t show any antitumor effect. LM3 cells incubated with Lip-LND could only kill very few cancer cells, which indicated that 15 µg mL^− 1^ LND might not be enough to induce efficient cell death. Due to the photodynamic and photothermal effects of IR780 under laser irradiation, many cancer cells were killed in “Lip-IR780 + L” group. Compared to the “Lip-IR780 + L” group, Lip-IR780/LND treatment with laser irradiation can lead to much more cell death. After treatment with Lip-IR780/LND under 808 nm laser irradiation, almost all of the LM3 cells were dead, which further indicated that LND could effectively enhance the therapeutic efficacy of IR780-induced photodynamic and photothermal therapy.

### In vivo evaluation of therapeutic effects of Lip-IR780/LND

**Fig. 5:**
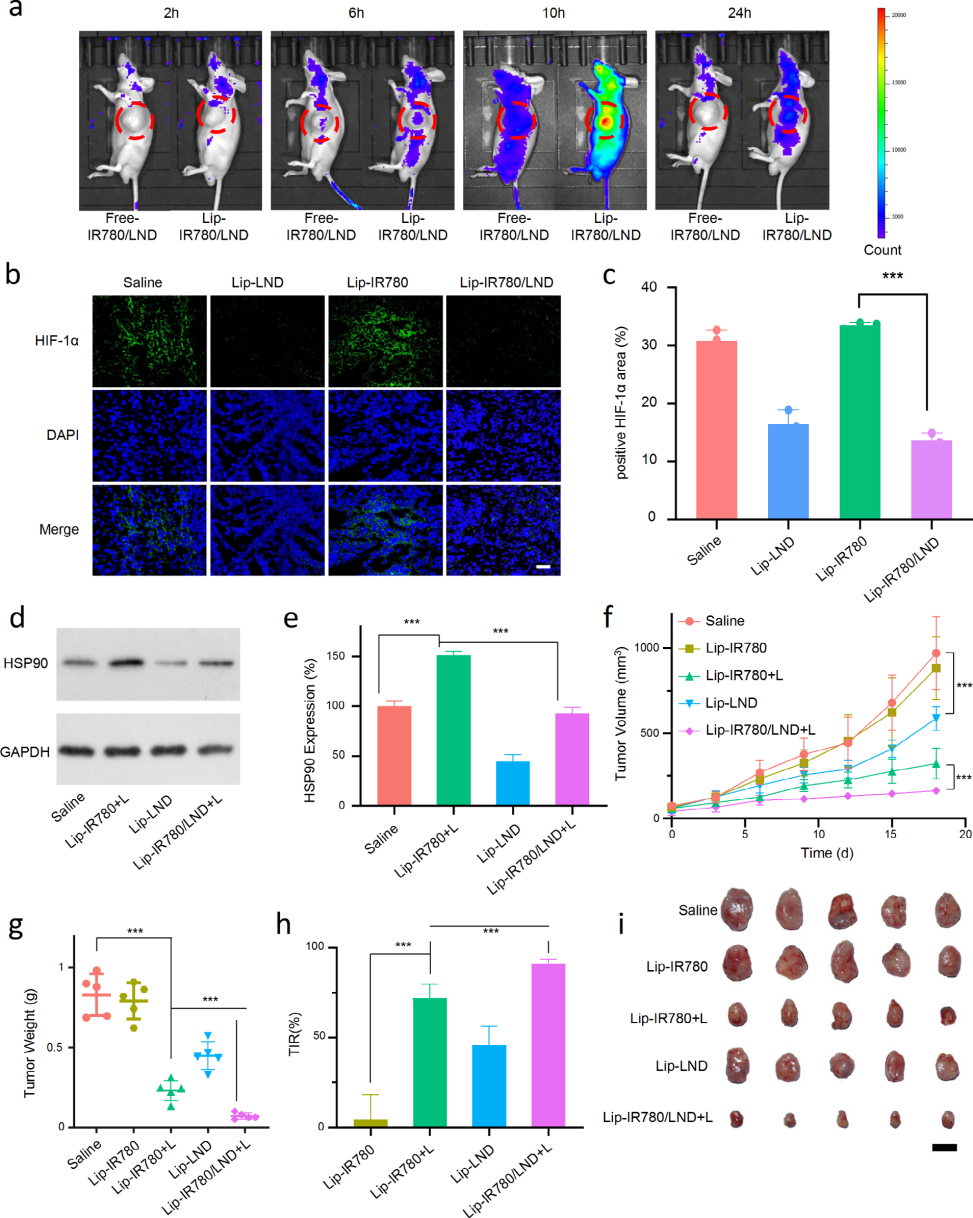
In Vivo Therapeutic Effect of Lip-IR780/LND. (**a**) Metabolism of free-IR780/LND and Lip-IR780/LND in the LM3 subcutaneous tumor model. (**b**) CLSM images of LM3 cells under HIF-1α kit after incubation with saline, Lip-LND, Lip-IR780, and Lip-IR780/LND. Scale bar: 50 μm. (**c**) The area of HIF-1α in different treatment groups (n = 3). (**d**) Western blot assay of HSP90 level after different treatments for 18 days. (**e**) Quantitative analysis of HSP90 expression (n = 3). (**f**) Tumor growth curves of LM3 tumor-bearing mice in different groups during the treatment period. (**g**) Changes in tumor weight in different treatment groups after 18 days (n = 5). (**h**) Tumor inhibition rate of each treatment group (n = 5). (**i**) Photographs of isolated tumors after 18 days of different treatments (n = 5). Scale bar: 1 cm. Data were expressed as means ± SD. ^*^p<0.05, ^**^p<0.01, ^***^p<0.001

Based on the excellent cell proliferation inhibition ability of LND enhanced phototherapy of IR780 in vitro, the anti-cancer performance of Lip-IR780/LND in vivo was further evaluated. Due to the excellent NIR fluorescent imaging capability of IR780, the in vivo biodistribution of drug-loaded liposomes was monitored in LM3-bearing nude mice after intravenous injection of Lip-IR780/LND. After Lip-IR780/LND or free-IR780 were intravenously injected into LM3-bearing nude mice, the in vivo biodistribution of IR780 was recorded by whole body fluorescent imaging after 2 h, 6 h, 10 h, and 24 h. As shown in Fig. 5a, a much stronger fluorescent signal of IR780 was observed in the tumor tissue of Lip-IR780/LND-treated mice than that of free IR780-treated mice. Therefore, Lip-IR780/LND could be effectively accumulated in tumor tissues, which was beneficial to the treatment of tumors. In addition, we explored the pharmacokinetics of liposomal drugs in mice.The tumor-free ICR mice were used to investigate the pharmacokinetics of Lip-IR780 after intravenous injection. As shown in Figure S3, the concentration of IR780 decreased to lower than 50% of the injected dose in plasma within 0.5 h in free-IR780/LND treated group. However, the blood circulation time of IR780 was significantly prolonged in Lip-IR780/LND-treated group, with the circulation half-life of 23.06 h.

Since LND could reduce oxygen consumption by inhibiting glycolysis, we further investigated whether LND can relieve tumor hypoxia in vivo. Hypoxia Inducible Factor 1 Alpha (HIF-1α) Kit and Hypoxyprobe-1 (HP-1) Kit were used to assess the level of hypoxia in tumor [45]. HIF-1α and HP-1 are two markers of tissue hypoxia, which can reflect the degree of hypoxia within the tumor tissues and can be observed by immunofluorescence staining [46, 47]. When the tumor tissues are at a hypoxic level, bright green fluorescence can be observed. The fluorescent intensity will decrease when the hypoxia is relieved. According to the fluorescent signal of HIF-1α in Fig. 5b and c, the level of hypoxia in tumor tissues could be effectively relieved after the mice were treated with Lip-LND and Lip-IR780/LND. However, Lip-IR780 cannot influence tumor hypoxia. Moreover, the relief of tumor hypoxia by Lip-LND and Lip-IR780/LND was also confirmed by hypoxic probe HP-1 based tissue section (Fig. S4 and S5) All these results demonstrated that LND could relieve tumor hypoxia by reducing oxygen consumption, which could provide more oxygen for IR780-triggered PDT.

During PTT in tumor therapy, the obvious temperature increase in tumor tissue was observed after 808 nm laser irradiation (Figure S6). Cytoprotective pathways of cancer cells can be activated under hyperthermia, leading to serious thermoresistance. HSPs are the key factor in thermoresistance, which are generally overexpressed under hyperthermia. The therapeutic effect of PTT can be improved by reducing the expression of HSP90. The expression of HSP90 in tumor tissues was then investigated by Western blot after different treatments. As shown in Fig. 5d and e, the expression of HSP90 in the Lip-IR780 treated group was significantly increased after laser irradiation, which implied that IR780-triggered PTT can lead to thermoresistance. Interestingly, Lip-LND treatment could remarkably reduce HSP90 level to 44.8% in tumor tissues, which implied that LND could overcome thermoresistance in PTT. Therefore, compared to phototherapy with IR780 (Lip-IR780 + L group), the expression of HSP90 was reduced to 61.2% after combined therapy with IR780-triggered phototherapy and LND. In conclusion, we demonstrated that LND could effectively reduce HSP90 expression to overcome thermoresistance in PTT, which was critical in improving the therapeutic efficacy of PTT.

The in vivo antitumor effect of Lip-IR780/LND under laser irradiation was then evaluated by tracking the tumor volume change after LM3-bearing nude mice were injected with Lip-IR780/LND. The LM3 tumor-bearing nude mice were randomly divided into five groups (five mice in each group) and injected with different formulations (saline, Lip-IR780, Lip-IR780 + L, Lip-LND, and Lip-IR780/LND + L). When the tumor volume reached about 100 mm^3^, Lip-IR780, Lip-LND, and Lip-IR780/LND were injected into the mice by tail vein at days 1, 3 and 5. After 6 h, the tumor tissues in Lip-IR780 + L and Lip-IR780/LND + L treated groups were irradiated with 808 nm laser for phototherapy. Monitoring of tumor volume showed that Lip-LND alone was not effective in inhibiting tumor growth. Lip-IR780 with laser irradiation can inhibit tumor growth. However, Lip-IR780/LND with laser irradiation had the best therapeutic efficacy. (Fig. 5f). 18 days after treatment, the mice were sacrificed. The tumor tissues were harvested and weighted. The weight of the tumors after treated with saline, Lip-IR780, Lip-IR780 + L, Lip-LND, and Lip-IR780/LND + L were 0.83 g, 0.79 g, 0.23 g, 0.45 g, and 0.07 g, respectively (Fig. 5g). The optimal therapeutic effect of Lip-IR780/LND + L was subsequently demonstrated by calculating the tumor inhibition rate. The tumor inhibition rate of Lip-IR780/LND + L reached 90%, which was significantly higher than that of other treatment groups (Fig. 5h). The above results demonstrated its favorable tumor-suppressive effect. Subsequently, in order to observe the tumor inhibition effect more directly, the dissected isolated tumors were photographed (Fig. 5i), and the results showed that Lip-IR780/LND + L treatment had the smallest tumor size, once again demonstrating its excellent anti-tumor effect in vivo.

To further obtain insight into the antitumor activity of Lip-IR780/LND under laser irradiation, the histologic sections of tumor tissues were analyzed by immunohistochemistry, including H&E staining and TUNEL assay. As expected, the highest apoptosis and lowest proliferation levels were observed in Lip-IR780/LND-treated tumor tissues after laser irradiation (Fig. S7). All these results above further indicated the excellent antitumor capability of Lip-IR780/LND under laser irradiation.

### Biosafety evolution in vivo

**Fig. 6:**
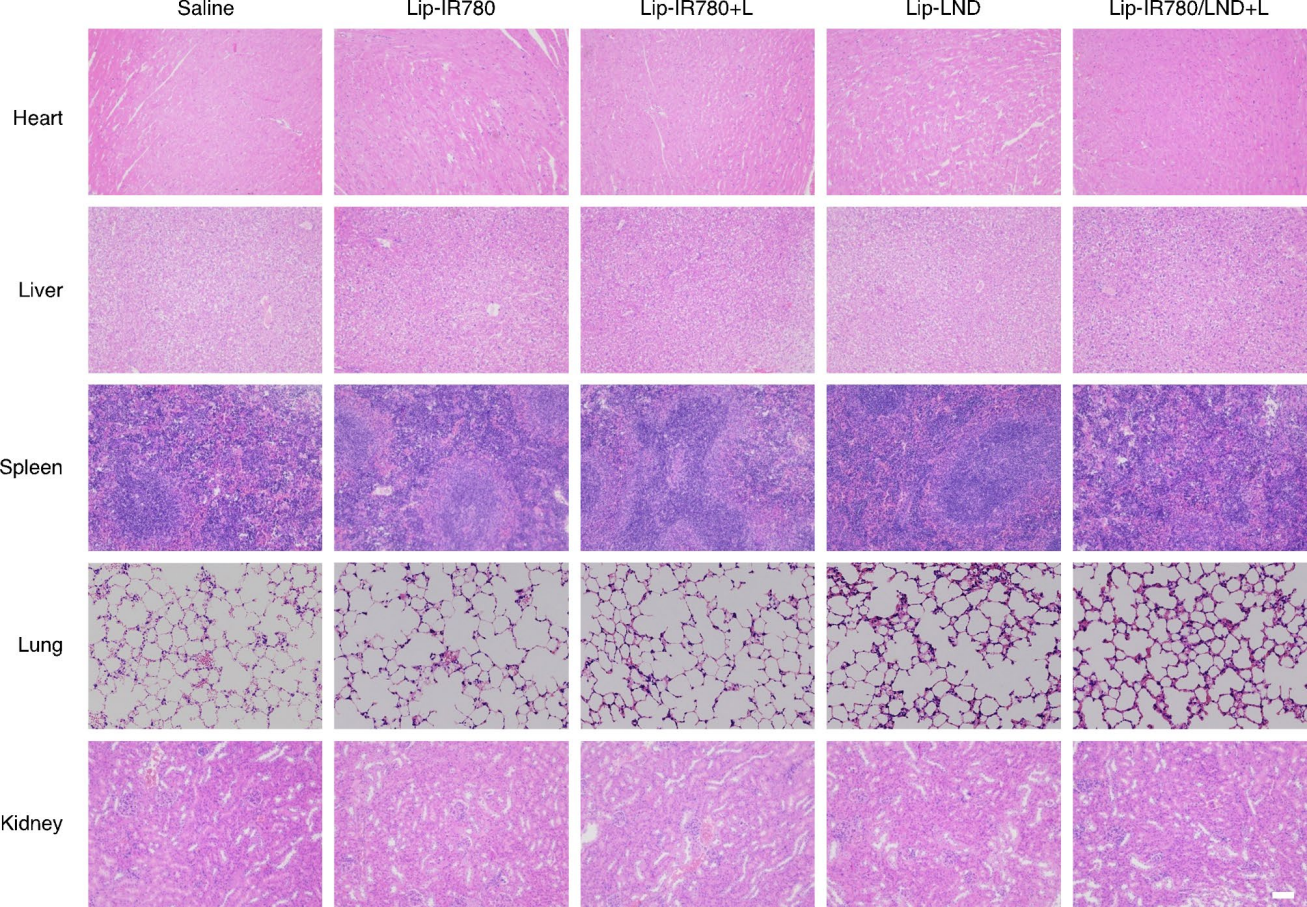
Biosafety of drug-loaded liposomes. H&E staining of Heart, liver, spleen, lung and kidney after 18 days of the corresponding treatment. Scale bar: 100 μm

The biosafety of the drug-loaded liposomes was finally evaluated. Firstly, we monitored the weight of the mice during the treatment period and did not find any significant weight changes (Fig. S8). Then, hematoxylin-eosin (H&E) staining of the heart, liver, spleen, lungs, and kidneys of mice after different treatments didn’t show significant damage or histopathological toxicity, demonstrating the biological safety of the liposomal drugs (Fig. 6). Finally, by assessing blood routine indicators including red blood cells, white blood cells (including the classification of white blood cells), platelets, and so on, no statistical differences were observed after different treatments (Fig. S9). All of these results indicated that the liposomal drugs showed excellent biosafety.

## Conclusion

In summary, we successfully prepared NIR probe IR780 and glycolysis inhibitor LND-loaded liposome Lip-IR780/LND for combined PDT/PTT. IR780 could perform both photodynamic therapy and photothermal therapy under the same light, while LND could effectively inhibit glycolysis after being taken up by LM3 cells. As an HK inhibitor, LND could effectively inhibit cellular respiration to reduce intracellular oxygen consumption to alleviate the hypoxic microenvironment of the tumor. Meanwhile, LND could also reduce intracellular GSH level. Therefore, more ROS can be generated in PDT. At the same time, LND could reduce energy supply by inhibiting glycolysis, which could down-regulate HSP90 level and therefore alleviate thermotolerance in tumor photothermal therapy. The in vivo results further confirmed the excellent tumor suppressive effects of LND enhanced. This study provides inspiration for future innovative designs of nanocarriers for enhanced photodynamic and photothermal therapy of hepatocellular carcinoma.

### Electronic supplementary material

Below is the link to the electronic supplementary material.

Supplementary Material 1

## Data Availability

The datasets used and analysed during this study are available from the corresponding author on reasonable request.
