# Supplementary material for: Lonidamine liposomes to enhance photodynamic and photothermal therapy of hepatocellular carcinoma by inhibiting glycolysis
Source: J Nanobiotechnology. 2023 Dec 15;21:482. doi: 10.1186/s12951-023-02260-z (PMC10724989; doi:10.1186/s12951-023-02260-z)

**Supporting information for:**

**Lonidamine liposomes to enhance photodynamic and photothermal therapy of** **hepatocellular carcinoma by inhibiting glycolysis**

Lei Lei^1†^, Wenbin Dai^2†^, Jiaping Man^2^, Haitao Hu^1^, Qiao Jin^2*^, Bo Zhang^3*^, Zhe Tang^1,3*^

^1^Department of Surgery, The Fourth Affiliated Hospital, International Institutes of Medicine, Zhejiang University School of Medicine, Yiwu, 322000, China

^2^MOE Key Laboratory of Macromolecule Synthesis and Functionalization of Ministry of Education, Department of Polymer Science and Engineering, Zhejiang University, Hangzhou, 310027, China

^3^Department of Surgery, The Second Affiliated Hospital, Zhejiang University School of Medicine, Hangzhou, 310058, China

E-mail: [jinqiao@zju.edu.cn](mailto:jinqiao@zju.edu.cn) (Q. Jin); [jjs10@zju.edu.cn](mailto:jjs10@zju.edu.cn) (B. Zhang); [8xi@zju.edu.cn](mailto:8xi@zju.edu.cn) (Z. Tang)

†These authors contributed equally

**Figure S1**: Confocal fluorescence micrographs of LM3 cells after incubation with Lip-LND, Lip-IR780, and Lip-IR780/LND for different periods of time. Scale bar :5 μm.

**Figure S2**: The expression of HSP90 after different treatments.

**FigureS3**: Pharmacokinetics profiles of IR780 in the tumor-free ICR mice after intravenous injection of IR780 formulations (n = 5).

**Figure S4**: CLSM images of LM3 cells under HP-1 kit after incubation with saline, Lip-LND, Lip-IR780, and Lip-IR780/LND. Scale bar: 50 µm.

**Figure S5**: The area of HP-1 in different treatment groups (n=3).


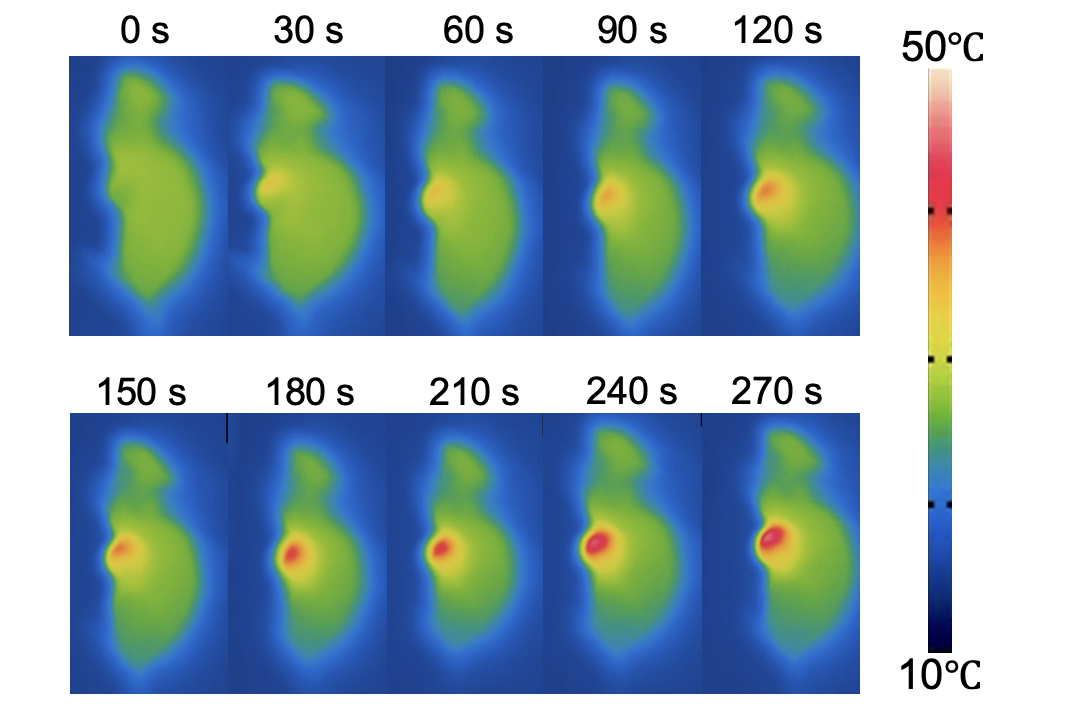


**FigureS6**: Near-infrared thermal imaging images of the tumor-bearing mice injected with Lip-IR780/LND and irradiated by 808 nm laser at 0.4 W cm^-2^.

**Figure S7**: The immunohistochemical analysis of tumor sections after different treatments by H&E and TUNEL. Scale bar: 100 μm.

**Figure S8**: The weights of LM3 tumors after isolated from nude mice at Day 18 after different treatments (n = 5).

**Figure S9**: Blood routine indicators after different treatment groups.

**Table S1**: The entrapment efficiency (EE) and drug loading content (DLC).


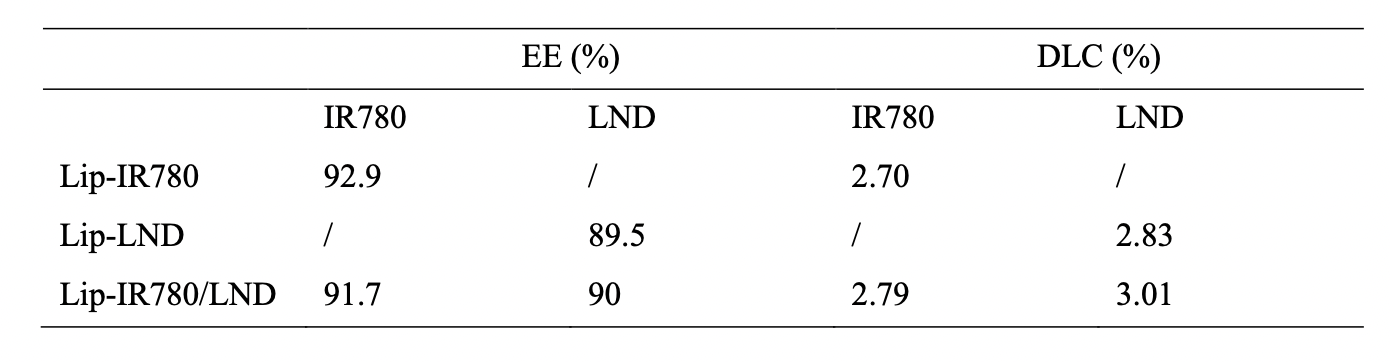

Supplement: Supplementary file 1 — Supplementary Material 1 [file 12951_2023_2260_MOESM1_ESM.docx]
